# Considerations in Liver Transplant Recipients Having Prior Roux-en-Y Gastric Bypass

**DOI:** 10.1097/TXD.0000000000001957

**Published:** 2026-05-12

**Authors:** Anita Mahadevan, James F. Crismale, Thomas Schiano, Lauren Tal Grinspan

**Affiliations:** 1 Department of Internal Medicine, Icahn School of Medicine at Mount Sinai, New York, NY.; 2 Recanati/Miller Transplantation Institute, The Mount Sinai Hospital, New York, NY.

Metabolic dysfunction–associated steatotic liver disease is the most rapidly growing indication for liver transplantation (LT) in the United States.^1,2^ Physicians are therefore more likely to care for patients with a history of bariatric surgery, both before and after LT. Twenty-two percent of bariatric surgery patients undergo Roux-en-Y gastric bypass (RYGB), which induces weight loss through mechanisms including malabsorption and restricted caloric intake.^3,4^ RYGB is rarely associated with sarcopenia, malnutrition, and hepatic injury that may require bypass reversal or LT.^5^

However, little is known regarding how prior RYGB may impact LT outcomes. Although studies have reported that pre-LT RYGB is associated with higher rates of nutritional deficiencies, sarcopenia, and mortality before transplant, some candidates ultimately undergo LT after RYGB.^6^ We sought to enhance current knowledge by describing 2 patients at our institution who underwent LT after RYGB and outlining the challenges faced, including rapidly progressive steatohepatitis, sarcopenia, and death. We outline how RYGB likely contributed to worsening clinical trajectory and review the complexities of posttransplant care for patients with a history of RYGB. Given that both patients had deceased, written informed consent was not obtained. This study did not meet the definition of human subjects research as it involves decedents only, is not a systematic investigation, and does not contribute to generalizable knowledge; therefore, Institutional Review Board review was not warranted.

## CASE DESCRIPTIONS

### Case 1

A 46-y-old woman with a remote RYGB underwent LT in 2021 for alcohol-related liver disease. Her initial immunosuppression (IS) regimen included cyclosporine, mycophenolate mofetil (MMF), and a prednisone taper. In the first 6 mo post-LT, her average cyclosporine level was 218 ng/mL; however, levels fluctuated significantly, ranging from 72 to 516 ng/mL. There were fluctuations even throughout hospitalizations, raising concerns about malabsorption of IS agents. Seven months after her transplant (March 2022), she developed elevated transaminases while still on cyclosporine and MMF, prompting a biopsy that demonstrated T cell–mediated rejection (TCMR), and she was treated with a steroid pulse and taper. Repeat liver biopsy (May 2022) demonstrated moderate TCMR requiring another pulse of steroids and optimization of maintenance IS with addition of sirolimus and discontinuation of MMF. Therapeutic IS levels were difficult to maintain, with many readmissions for undetectable levels despite confirmed medical compliance, raising concern about malabsorption. During these admissions, she was noted to have significant malnutrition and required tube feeds. Fecal studies were notable for fecal elastase <50 µg/g and normal calprotectin, and she was found to have an l-carnitine deficiency. Her course was complicated by ongoing chronic ductopenic rejection treated with steroids and belatacept (October 2022) and antibody-mediated rejection treated with plasmapheresis, IVIG, and rituximab (December 2022). After several readmissions resulting in graft failure and encephalopathy, she underwent repeat LT 23 mo after her first LT.

Her IS regimen after her second transplant included tacrolimus, MMF, and prednisone. Her subsequent post-LT course was complicated by persistent hypoglycemia and recurrent admissions for renal injury, weight loss with severe malnutrition, undetectable tacrolimus levels, and sepsis. Her tacrolimus levels fluctuated, including while hospitalized under controlled conditions. Micronutrient levels, including copper, selenium, and vitamins A, D, and B6, which were not included in standard pretransplant assessments, were low. She required prolonged enteral nutrition. Liver biopsies were notable for severe steatohepatitis (September 2023) and then, severe steatohepatitis with evidence of moderate TCMR (December 2023). The severe steatohepatitis was thought to be caused by malnutrition due to her prior RYGB; all testing for surreptitious alcohol use was negative. She developed worsening graft dysfunction and expired within the first year after her second LT.

### Case 2

A 55-y-old woman with a remote RYGB with omega loop received LT in March 2022 for metabolic dysfunction–associated steatotic liver disease. Biliary reconstruction was performed via Roux-en-Y hepaticojejunostomy, bypassing additional foregut, and her initial IS regimen included tacrolimus, MMF, and prednisone. She was hospitalized 1 mo post-LT with diarrhea and intolerance to oral intake, which resolved with supportive care and substituting her MMF with mycophenolic acid. Four months post-LT, she was readmitted with *Clostridium difficile* and cytomegalovirus viremia infections. She was empirically treated for small intestinal bacterial overgrowth. She required both enteral nutrition and parenteral nutrition for several months. Push enteroscopy and colonoscopy, including mucosal biopsies, were overall unremarkable, and additional fecal studies were not performed. Liver biopsy performed to evaluate elevated aminotransaminases demonstrated severe steatosis (90%) and stage 3 fibrosis; the donor liver graft had no preexisting fibrosis. Her tacrolimus levels in the 7 mo following transplant fluctuated despite medical compliance. Testing revealed deficiencies of essential fatty acids, such as linoleic and alpha-linolenic acids, as well as zinc, manganese, and vitamins A, D, B6, and C, many of which were not tested as part of her standard pretransplant nutritional evaluation.

The patient developed anasarca in the setting of profound hypoalbuminemia and oliguric kidney failure requiring hemodialysis. She developed progressive allograft dysfunction, thought to be due to intestinal failure-associated steatohepatitis based on her previous biopsy findings. RYGB reversal was considered; however, she was determined not to be a candidate due to critical illness, and she expired 15 mo post-LT.

## DISCUSSION

Here we discuss 2 cases of patients with prior RYGB who underwent LT with posttransplant complications, including malnutrition, micronutrient deficiencies, sarcopenia, severe steatosis, and death. This patient population has not been widely studied, and although prior investigations have proposed that RYGB is not associated with any difference in LT outcomes, we propose that bypass anatomy in these cases contributed significantly to their complications.^1,7^

Malnutrition and rapid weight loss are linked to the development of severe steatohepatitis.^8^ Our patients had many of these complications posttransplant, including rapidly progressive steatosis and fibrosis, suggesting that patients with prior RYGB may be at higher risk for recurrent steatosis or fibrosis after LT. Bypass reversal to rescue the liver and progression to LT have both been reported.^9,10^ In patients who are post-LT with a history of RYGB and face the development of severe steatohepatitis, bypass reversal may salvage graft function.^11^ The length of gut bypassed is an important factor regarding the severity of complications. Historically, jejunoileal bypasses, which were performed in the 1970s, were associated with malnutrition, hepatic steatosis, and rapidly progressive fibrosis and often had to be reversed.^12-14^ Similarly, omega loops are known to pose challenges, including malabsorption and afferent loop syndrome.^15^

Malabsorption of IS is a crucial consideration in post-LT patients with RYGB. The current literature on IS absorption in post-LT patients with RYGB is limited, with more published data in kidney transplant patient populations. A pilot study of 6 patients receiving hemodialysis or status post–renal transplant concluded that due to pharmacokinetic changes in patient status post–gastric bypass surgery, transplant recipients would likely require higher doses of immunosuppressants.^16^ However, several studies, including a case report of a patient who underwent orthotopic LT after RYGB, showed that no changes in IS were needed in patients with several different forms of bariatric surgery.^17^ However, the current patient cases suggest that IS absorption may be altered in patients with prior RYGB. Even in a controlled hospitalized setting, our first patient’s IS management proved challenging due to fluctuating trough levels. Considering both patients’ several nutritional deficiencies as well, we believe that malabsorption is the common denominator. Physicians should be aware of possible IS malabsorption, especially in patients with other signs of malabsorption, such as nutrient deficiencies and fluctuating IS levels.

Patients with prior RYGB should be nutritionally optimized before transplant. Our patients were found to have several micronutrient deficiencies after LT that are not typically evaluated for during a standard pretransplant nutritional assessment. Rigorous nutritional examination and micronutrient deficiency screening should be performed in these patients, and deficiencies should be aggressively addressed with supplementation—especially in patients with long segments of bypassed intestine (Table 1). If feasible, performing a duct-to-duct anastomosis for biliary reconstruction at the time of LT may be preferable to hepaticojejunostomy to avoid bypassing further intestine.

**TABLE 1.** Pretransplantation micronutrient screening recommendations in patients with prior RYGB

Micronutrient or element	Clinical relevance
Selenium	RYGB is associated with an increased risk of selenium deficiency.^18^ Deficiency is linked to sarcopenia^19^
Copper	RYGB can cause copper deficiency, which has been shown to be associated with a higher rate of pretransplant mortality^18,20,21^
Iron	Iron deficiency is seen frequently following RYGB, and deficiency is associated with increased posttransplantation mortality^20,22^
Zinc	Zinc deficiency may be seen both after RYGB and in patients with chronic liver disease.^20,23^ Pretransplant, screening for zinc deficiency should be pursued with the intent to supplement if deficient; however, following transplantation, levels have been shown to normalize^23^
Vitamins A, D, B_1_, B_6_, and B_12_	RYGB has been associated with deficiency in all these nutrients and routine testing should be performed to correct any deficiencies^20^

RYGB, Roux-en-Y gastric bypass.

Ultimately, for patients with higher-risk features, such as sarcopenia, which has been associated with morbidity and mortality post-LT, an omega loop, or longer Roux limb length, surgical revision may be considered.^24^ Although this was ultimately not pursued in our patients, we propose that bariatric surgery consultation should be considered for evaluation of surgical takedown as a preventative measure or for patients facing possible retransplantation.

## CONCLUSIONS

We present 2 cases in LT recipients where prior RYGB likely contributed to malnutrition/malabsorption, severe steatohepatitis, and death. Although a sleeve gastrectomy is undoubtedly preferred in current practice to reduce risk of nutritional deficiencies and further complications, a significant portion of patients presenting for LT have a history of a RYGB. Physicians should be aware that this cohort of patients may face an increased risk of malabsorption, recurrent steatosis and fibrosis, sarcopenia, and nutritional deficiencies after transplantation. Risk stratification should include consideration of the length of bypassed limb and expanded preoperative nutritional evaluation, with consideration of bypass reversal if high risk. Postoperatively, it is necessary to regularly test for micronutrient deficiencies and monitor for signs of steatosis and fibrosis, as timely intervention is necessary to provide adequate supplemental nutrition to prevent graft loss and death.
